# Evaluating the efficacy of an online, family-based intervention to promote adolescent sexual health: a study protocol for a randomized controlled trial

**DOI:** 10.1186/s13063-023-07205-3

**Published:** 2023-03-11

**Authors:** Vincent Guilamo-Ramos, Adam Benzekri, Marco Thimm-Kaiser

**Affiliations:** 1 Center for Latino Adolescent and Family Health, Duke University School of Nursing, Durham, NC USA; 2 School of Nursing, Duke University, 307 Trent Dr, Durham, NC 27710 USA; 3 School of Medicine, Department of Family Medicine and Community Health, Department of Infectious Diseases, Duke University, 40 Duke Medicine Circle, Durham, NC USA; 4 Presidential Advisory Council on HIV AIDS, U.S. Department of Health and Human Services, Washington, D.C., USA

**Keywords:** Sexual and reproductive health, Adolescents, Parents, Unprotected sex acts, Teen pregnancy, STI, HIV

## Abstract

**Background:**

Adolescents in the U.S. experience significant negative sexual health outcomes, representing a public health priority in the U.S. Research shows that while parents play an influential role in shaping adolescent sexual behavior, surprisingly few programs engage parents in existing programming. Moreover, most efficacious parent-based programs focus on young adolescents, and few utilize delivery mechanisms that facilitate broad reach and scale-up. To address these gaps, we propose to test the efficacy of an online-delivered, parent-based intervention adapted to address both younger and older adolescent sexual risk behavior.

**Methods:**

In this parallel, two-arm, superiority randomized controlled trial (RCT), we propose to evaluate Families Talking Together Plus (FTT+), an adaptation of an existing and efficacious FTT parent-based intervention, in shaping sexual risk behavior among adolescents aged 12–17 and delivered via a teleconferencing application (e.g., Zoom). The study population will include *n*=750 parent–adolescent dyads recruited from public housing developments in the Bronx, New York. Adolescents will be eligible if they are between the ages of 12 and 17 years of age, self-report as Latino and/or Black, have a parent or primary caregiver, and are South Bronx residents. Parent–adolescent dyads will complete a baseline survey, after which they will be assigned to either the FTT+ intervention condition (*n*=375) or the passive control condition (*n*=375) in a 1:1 allocation ratio. Parents and adolescents in each condition will complete follow-up assessments 3 and 9 months post-baseline. The primary outcomes will include sexual debut and ever sex, and the secondary outcomes will include the frequency of sex acts, number of lifetime sexual partners, number of unprotected sex acts, and linkage to health and educational/vocational services in the community. We will utilize intent-to-treat analyses of 9-month outcomes and single degree of freedom contrasts comparing the intervention to the control group for primary and secondary outcomes.

**Discussion:**

The proposed evaluation and analysis of the FTT+ intervention will address gaps in the current cadre of parent-based programs. If efficacious, FTT+ would represent a model for scale-up and adoption of parent-based approaches designed to address adolescent sexual health in the U.S.

**Trial registration:**

ClinicalTrials.gov NCT04731649. Registered on February 1, 2021.

## Administrative information

**Table:** 

Scientific title {1}	Evaluating the efficacy of an online, family-based intervention to promote adolescent sexual health: a study protocol for a randomized controlled trial
Trial registration {2a and 2b}.	ClinicalTrials.gov (NCT04731649)
Protocol version {3}	Last updated: July 15, 2022
Funding {4}	Research reported in this publication was supported by the Administration for Children and Families under Award Number 90SR0113-01-00 (Principal Investigator: VGR).
Author details {5a}	^1^Center for Latino Adolescent and Family Health, Duke University School of Nursing, Durham, North Carolina, United States ^2^School of Nursing, Duke University, 307 Trent Dr, Durham, NC 27710, United States ^3^School of Medicine, Department of Family Medicine and Community Health, Department of Infectious Diseases, Duke University, 40 Duke Medicine Circle, Durham, North Carolina, United States ^4^Presidential Advisory Council on HIV AIDS, U.S. Department of Health and Human Services, Washington, D.C., United States
Name and contact information for the trial sponsor {5b}	Duke University, Durham NC, 27708, U.S.
Role of sponsor {5c}	The sponsor is responsible for the study design, collection, management, analysis and interpretation of data, writing of the report, and decision to submit the report for publication.
Contact for public queries	Adam Benzekri, Intervention Coordinator, 407-852-9332, ab7170@nyu.edu
Contact for scientific queries	Principal Investigator: Vincent Guilamo-Ramos, Vincent.ramos@duke.edu, 919-684-9444

## Introduction

### Background and rationale {6a}

Adolescents in the U.S. face significant, and in some instances worsening, negative sexual and reproductive health (SRH) outcomes [1–6], which represent a public health priority in the U.S. [3, 5, 7].

While the number of annual teen pregnancies has declined by 64% in the past two decades, teen pregnancy rates in the U.S. are among the highest of all developed countries [1, 2]. Since 2013, sexually transmitted infection (STI) incidence is well above historical levels for youths aged 15 to 24 [4, 5]. Notably, youth under the age of 25 accounted for 55% of all new reported cases of STIs [4] and approximately 20% of all new cases of HIV in 2020 [6] despite comprising only about 13% of the population [8]. Latino and Black youth are disproportionately represented in these figures, accounting for approximately four in five new annual diagnoses of HIV among youth, approximately half of annual youth STI cases, and significantly higher rates of teen pregnancies and births relative to overall rates [2, 4, 6]. This is of concern, particularly given significant progress in reducing sexual activity among adolescents in the U.S. in the past three decades [9].

Since 1991, the proportion of adolescents who report ever having sexual intercourse has decreased by nearly 30% [9]. Data suggest that while the average age of sexual debut has increased [9, 10], the proportion of older adolescents who report sexual activity has remained much more stable, only decreasing by approximately 15% since 1991 [9]. Furthermore, older adolescents aged 17 to 19 represent approximately 90% of annual births among adolescents aged 10 to 19 [2]. Similarly, among youth in the U.S., the risk of STI and HIV diagnosis increases with age [4–6], suggesting that older adolescents are in particular need of efficacious sexual health programming. Novel approaches are needed in the prevention of negative sexual health outcomes among older adolescents.

A recent report from the National Academies of Sciences, Engineering, and Medicine (NASEM) highlights the important role of parents in shaping adolescent sexual health [5]. Specifically, parent–adolescent communication about sex is associated with delayed sexual initiation, reduced number of sexual partners, and increased condom use [11–14]. Parental monitoring, such as parents setting rules about dating with their adolescents and being aware of their adolescents’ whereabouts when they are not in the home, is associated with reduced risk of pregnancy and delayed sexual initiation [15, 16]. In addition, adolescents consistently report their parents as the most important influence in their decisions about sex [17].

While parent-based approaches to addressing adolescent sexual health exist, two gaps stand out: (1) most efficacious programs focus primarily on young adolescents; and (2) few utilize delivery mechanisms that facilitate broad reach and scale-up. First, the majority of extant parent-based programs are designed for and efficacious among younger adolescents [18]. In a systematic review and meta-analysis, age moderated the association of parent-based interventions on adolescent sexual health outcomes, with larger effect sizes in studies with developmentally younger age groups [18]. The scarcity of efficacious parent-based interventions in shaping older adolescent sexual health is particularly striking, given (1) the increasing age of sexual debut in the U.S.; (2) reductions in parental monitoring and communication about sex that occur with age; and (3) reports from older adolescents of wanting more parental guidance in regard to their sexual health and decision-making [9, 10, 19]. There is a need for the developmental adaptation, evaluation, and dissemination of existing, efficacious parent-based SRH interventions, such as FTT, to address older adolescents’ sexual health outcomes. Second, the reach of extant parent-based programs designed to address adolescent sexual health remains inadequate [20]. Development and evaluation of novel delivery models are sorely needed to facilitate greater intervention scale-up and reach. Remote/virtual delivery models using teleconferencing applications (e.g., Zoom) represent one such novel and promising approach. During the COVID-19 pandemic, similar delivery models have been successfully adopted at scale for the delivery of a variety of health services and prevention programs, ranging from therapeutic services to sexual health [18, 21, 22]. Taken together, the existing cadre of parent-based programming is not well aligned with changing patterns of adolescent sexual activity and outcomes, and the reach of existing programs remains inadequate. The adaptation, evaluation, and scale-up of existing programs in the prevention of older adolescent sexual health negative outcomes are needed. Families Talking Together (FTT) represents one such program that uniquely focuses on parents in the reduction of adolescent sexual risk behavior, unplanned pregnancies, and STIs.

FTT has been shown to be efficacious in multiple large-scale randomized controlled trials (RCTs) in delaying sexual debut, increasing condom use, and reducing the frequency of sex among adolescents aged 10 to 14 within a range of settings (i.e., health clinics, schools, community-based organizations, and households) [23–25]. Based on this evidence, FTT has been identified by the U.S. Department of Health and Human Services (HHS), the U.S. Preventive Services Task Force, the American Academy of Nursing, and NASEM as a rigorously evaluated, evidence-based, and efficacious sexual health program [5, 26–28].

In this parallel, two-arm, superiority RCT, we propose to evaluate Families Talking Together Plus (FTT+), an adaptation of the existing and efficacious FTT parent-based intervention. FTT+ is adapted for delivery to parents with adolescents aged 12–17 utilizing a remote/virtual delivery platform. If efficacious, FTT+ would represent a model for scale-up and adoption of parent-based approaches designed to address adolescent sexual health.

### Objectives {7}

The primary objective of the proposed study will be to evaluate the efficacy of the FTT+ intervention in reducing adolescent sexual risk behaviors, including engagement in vaginal, anal, and oral sexual intercourse, debut of vaginal, anal, and oral sexual intercourse, and number of unprotected sex acts.

### Trial design {8}

**Fig. 1:**
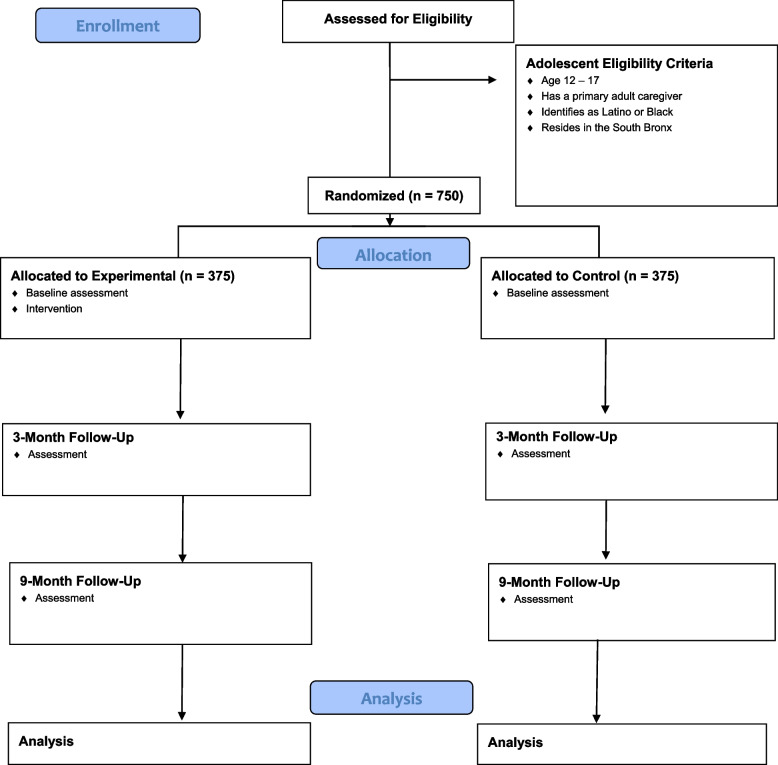
Families Talking Together Plus: consort diagram

The proposed study is a parallel, two-arm, superiority RCT designed to evaluate the efficacy of FTT+ in shaping sexual risk behavior among adolescents aged 12–17 delivered via a remote/virtual teleconferencing platform. We will recruit and enroll 750 parent–adolescent dyads from public housing developments in the South Bronx, New York City (NYC). Data collected for this study will consist of baseline and follow-up assessments via self-report surveys. After the baseline assessment, each enrolled dyad will be randomly assigned to either the experimental (*n*=375) or the passive control (*n*=375) arm in a 1:1 ratio. In the experimental arm, parents will receive FTT+ intervention sessions with a community health worker (CHW) delivered via a teleconferencing application (e.g., Zoom). Participants in the passive control group will not receive any intervention following the baseline. All study participants will complete follow-up surveys 3 and 9 months post-baseline. The study is registered with ClinicalTrials.gov (NCT04731649). A CONSORT flow diagram of the study activities is shown in Fig. 1.

## Methods: participants, interventions, and outcomes

### Study setting {9}

The primary site of recruitment and data collection will be the South Bronx. The South Bronx is a socioeconomically and medically underserved community that represents a context of increased exposure and susceptibility to negative sexual health outcomes. It is home to the poorest congressional district in the continental U.S. [29], is a Health Resources and Services Administration (HRSA)-designated medically underserved area [30], and has higher rates of STIs, HIV, and teen pregnancy than both national and city levels [31]. The current study will collect survey data in either the participants’ homes or a mutually agreed upon location in the community. Intervention delivery, however, will occur remotely through a teleconferencing application (i.e., Zoom) from the Center for Latino Adolescent and Family Health (CLAFH) Manhattan office.

### Eligibility criteria {10}

Dyads will be eligible to participate in this study if the adolescent is Latino and/or Black, between the ages of 12 and 17, resides in the South Bronx, and reports having a parent. A parent will be defined for the purposes of this study as the adolescent’s biological or nonbiological primary adult caregiver. Adolescents will be excluded if they are neither Latino and/or Black, younger than 12 or older than 17 years of age, report not having a parent, or do not reside in the South Bronx.

### Who will take informed consent? {26a}

If dyads are eligible to participate, data collectors will conduct the consent/assent interview either in person or over the phone. Parents will provide both verbal consent and permission for their own and their adolescents’ participation, respectively. Meanwhile, adolescent minors will provide their verbal assent at baseline and will be asked to provide their consent if they turn 18 during the course of the study. Formal recruitment and consent/assent interviews will occur in either English or Spanish, depending on participant preference.

### Additional consent provisions for collection and use of participant data and biological specimens {26b}

All data will be retained for 6 years after the study is completed or until the adolescent reaches the age of 21, whichever is longer. Retaining data for this period of time is required by the Duke University Health System for regulatory purposes. Participants’ data and records may be reviewed in order to meet federal or state regulations, as per the Consent to Participate in a Research Study agreement. A review may be performed by representatives of Duke University School of Nursing (DUSON), the Administration for Children and Families (ACF) representatives and affiliates, the Duke University System Institutional Review Board (IRB), and/or others as appropriate. We will not obtain biological specimens.

## Interventions

### Explanation of the choice of comparators {6b}

The trial will compare the efficacy of the FTT+ intervention to a passive control group. We hypothesize that adolescents whose parents are enrolled in the FTT+ intervention will report fewer sexual risk behaviors than adolescents whose parents are in the control condition, including engagement in vaginal, anal, and oral sexual intercourse, debut of vaginal, anal, and oral sexual intercourse, and number of unprotected sex acts. In addition, we hypothesize that adolescents in the experimental group will be more likely to report linkage to health and educational/vocational services in the community than those in the passive control group.

### Intervention description {11a}

#### Intervention condition

FTT+ is an online intervention designed to reduce adolescent sexual risk behavior through supporting caregiver–adolescent communication about sexual risk reduction (e.g., delayed sexual debut, reduced frequency) and condom use.

The FTT+ intervention consists of four components: (1) face-to-face sessions with FTT+ CHWs delivered remotely; (2) a written FTT workbook; (3) supplemental FTT communication aids for adolescents and parents; and (4) FTT life opportunity resources. A detailed outline of each component of the FTT+ intervention and the content covered is given in Table 1, below.

**Table 1:** Outline of each component of the FTT+ intervention and the content covered

**Component**	**Topics covered**
Component 1: Face-to-face sessions with FTT+ CHWs delivered remotely Parents will receive face-to-face sessions, via Zoom, with FTT+ CHWs. CHWs will deliver the intervention content personalized to the family’s community and context, offer the opportunity to practice newly acquired skills, and serve as a resource for addressing questions and concerns.	• Parent-specific framing of content: CHWs will frame intervention content personalized to the family, community, and local health context. • Collaboration and scaffolding: Parents and CHWs will collaborate on the understanding of the material. Parents will role-play and receive feedback from the CHWs to ensure knowledge and skills for effective parent–adolescent communication. • Inquiry-based learning: CHWs will address questions and concerns and use questions as a guide to framing the intervention content to the needs of specific parents. • Deliberate pacing of materials: Discussion of FTT materials is paced throughout each session to allow for parents to learn the information in chunks, rather than at once. • Reciprocity and responsiveness: Parents will feel more inclined to actively engage with the material when a CHW reviews the content with them and encourages parent participation throughout the intervention sessions.
Component 2: Written FTT workbook Parents will receive the written FTT workbook containing the entire FTT content organized in nine modules.	• Accessibility of intervention content and sexual risk avoidance resources: Complete FTT+ intervention content, structured and readily available at all times. • Ongoing parent support: The FTT+ workbook will serve as a resource to answer questions, clarify intervention session content, and provide guidance and support to parents who encounter problems implementing FTT+ parenting strategies.
Component 3: Supplemental FTT communication aids for adolescents and parents FTT+ will contain communication aids that are designed for use at home to facilitate parent–adolescent communication about delaying sex and promoting correct and consistent condom use	• Adolescent materials for resource-based learning: Adolescents will receive two booklets that are designed for joint use by both parents and adolescents and contain key intervention messages regarding waiting to have sex as well as correct and consistent condom use. • Summary for parents to reinforce key intervention content: Parents will receive the short “The Basics” booklet that is designed to serve as a condensed resource to consolidate the key points of the intervention content and guide discussions with adolescents. • Homework assignments: CHWs will assign parent–adolescent dyads specific activities and exercises using the communication aids to reinforce FTT+ core content and provide shared activities to further understand and integrate the material in their daily lives.
Component 4: FTT+ life opportunity resources The FTT+ intervention will contain specific content designed to support adolescent goal setting, success sequencing, and linkage to health and educational/vocational services in their community	• Establishment of long-term goals: CHWs will emphasize the importance of parent–adolescent discussions about setting long-term goals in relation to avoiding sexual risk behaviors and correctly using a condom every time if sexually active, as well as adolescent life opportunities. • Support for success sequencing: FTT+ intervention reading materials will provide guidance for effective parenting strategies to promote delaying sexual activity and correct and consistent condom use. • Linkages to community-based health and educational/vocational resources: CHWs will encourage engagement in community­ specific resources and services that are available to adolescents. The FTT+ packet will provide a directory of free educational and tutoring opportunities as well as vocational training and internship sites.

#### Intervention delivery

For FTT+ intervention delivery and family linkage to community social services and resources, the proposed project will rely on CHWs, who are embedded in the target community and who will be trained to deliver the program. CHWs have historically worked in communities underserved by mainstream healthcare systems and are effective in reaching populations who may otherwise lack access to healthcare services. Because CHWs are indigenous to the communities they serve, they are uniquely positioned to deliver healthcare information and services in a culturally relevant and linguistically appropriate fashion. As such, CHWs will address some of the primary access barriers to health education and services encountered by individuals in underserved communities such as within the South Bronx. All CHWs will be certified as FTT+ interventionists and receive ongoing training in delivering the FTT+ intervention with fidelity.

The FTT+ intervention sessions will be conducted over a teleconferencing application (e.g., Zoom) in either English or Spanish, depending on the participant’s preference. CHWs will familiarize parents with the written FTT+ curriculum materials; motivate parents to talk with their adolescent; directly address some of the key factors our research found to be associated with adolescent sexual activity; stress the importance of establishing expertise and trust and being accessible; review the communication aids and life opportunities resources; and emphasize issues in monitoring and establishing connectedness. FTT+ materials will be provided to participating families in-person immediately following completion of their baseline surveys and opening of the allocation envelope. Each of the remote intervention delivery sessions will last approximately 45 min.

### Criteria for discontinuing or modifying allocated interventions {11b}

The primary criteria for discontinuing the allocated intervention would be based on experimental family request to no longer receive the FTT + intervention. There are no plans to modify allocated interventions.

### Strategies to improve adherence to interventions {11c}

The scheduling of intervention sessions will be flexible, and appointments will be planned around participant availability, including during evening hours and on weekends. Interventionists will call an hour before the appointment to remind the participant of their scheduled intervention. To ensure high fidelity of intervention delivery, the interventionist coordinator will review recordings of at >10% of all intervention sessions performed by CHWs. This practice will allow for continuous re-training of the CHWs, who will be able to apply the feedback they receive to optimize intervention fidelity and dosage.

### Relevant concomitant care permitted or prohibited during the trial {11d}

There are no restrictions on the healthcare programs in which participants partake.

### Provisions for post-trial care {30}

Trial participation presents no more than minimal risks to participants. Therefore, no provisions for post-trial care are needed.

### Outcomes {12}

The primary outcomes of interest will be assessed at the delayed (9-month) follow-up survey.

1. Sexual debut/Ever sex (vaginal, anal, oral sex)

Ever sex will be assessed using three items of lifetime sexual activity. Adolescents will be asked whether they have ever had (1) vaginal, (2) anal, or (3) oral sex in their lifetime. To assess sexual debut, we will create a dichotomous outcome using baseline and 9-month data: 1 = adolescent reported ever having sex at 9-month follow-up and not at baseline and 0 = adolescent reported ever having sex at baseline or never having sex at 9-month follow-up.

The secondary outcomes of interest are four measures of adolescent SRH:

1. Frequency of sex (vaginal, anal, oral sex)
2. Number of sexual partners
3. Number of unprotected sex acts (vaginal, anal, oral sex)
4. Linkage to health and educational/vocational services.

The frequency of sex will be assessed using three items. At the 9-month follow-up, participants will be asked the number of times they have had (1) vaginal, (2) anal, or (3) oral sex in the past 9 months.

Number of lifetime sexual partners will be assessed using one item. At the 9-month follow-up, we will ask the participant how many different people they have had sexual intercourse with.

The number of unprotected sex acts will be assessed using three items. We will ask participants to report the number of times they have had (1) vaginal, (2) anal, or (3) oral sex without using a condom in the past 9 months.

Lastly, adolescent linkage to health and educational/vocational services in the community will be assessed using two measures. The participant will be asked which community resources they have accessed in the past 9 months out of a range of options, after which they will be asked about their intentions to access some of these same community resources in the coming 6 months. This will be scored using the Utilization of Community Resources Scale, with higher scores indicating greater linkage to services in the community addressing current needs (e.g., food insecurity) or economic capital (e.g., job training): 0 signifies no linkage to any services listed, and 1 signifies linkage to all types of services listed in the scale.

### Participant timeline {13}

All participants will be asked to complete three questionnaires at different time points to assess the outcomes of interest. The first questionnaire is the baseline assessment, used to establish group equivalence across key variables prior to randomization. Importantly, experimental and control groups are hypothesized to be similar enough to attribute any significant differences in outcomes to the intervention and not to other variables. Baseline assessments will be given at time of enrollment, after the participant has been screened for eligibility and provided their consent/assent to participate. The second questionnaire will be administered 3 months post-baseline assessment. At the 3-month follow-up, we hypothesize that we will be able to detect changes to adolescent reports of mediator variables (e.g., parental monitoring and supervision, decision-making) between the intervention and control groups. The third questionnaire will be administered 9 months after the baseline assessment and will be the primary point of contrast between the experimental and control groups in the primary and secondary outcomes. The timeline of the delayed follow-up was chosen based on the developmental age of the sample and as an appropriate timeframe to detect adolescent sexual behavior, which is often characterized as sporadic and short-lived. Table 2 below provides the timeframe from enrollment to post-allocation along with the outcomes to be measured at the 9-month follow-up.

**Table 2:**
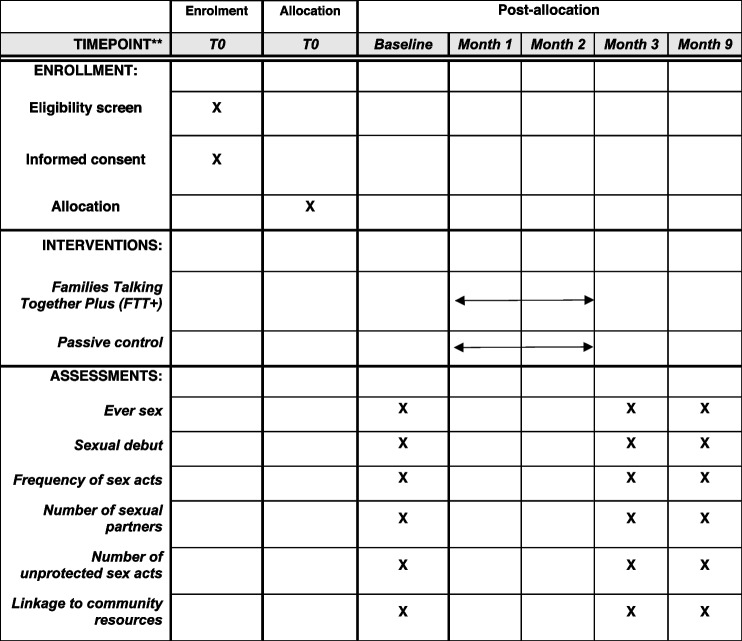
Timeframe from enrollment to post-allocation

### Sample size {14}

We will enroll 750 parent–adolescent dyads, allocated to either the FTT+ intervention condition or the passive control condition in a 1:1 allocation ratio. For single degree of freedom contrasts of means, a sample size of approximately 337 per group yields a power of 0.80 to detect a minimal detectable impact (MDI) of .9. For comparison of percentages, a sample size of approximately 337 per group yields a power of 0.80 to detect a 9% difference between two groups when the population percentages are in the ranges observed in our previous research [23–25]. In prior studies with passive control groups that evaluated the impact of FTT, the primary outcome differed by >9% between arms. The proposed study includes 375 dyads per group to accommodate attrition over the course of the project (expected to be low, <20% overall attrition, <5% differential attrition) and to permit sensitivity analyses (i.e., Latino vs. Black, males vs. females, younger vs. older adolescents).

### Recruitment {15}

The primary recruitment strategy of FTT+ will be area sampling via door-to-door recruitment. This form of face-to-face contact will enable our project to directly engage with community members and inform them of our work to address sexual health, link adolescents to community resources, and support the health and well-being of adolescents in the South Bronx more broadly. Participating families will be recruited by trained recruiters. Recruiters will canvass public housing developments in the South Bronx using area sampling methods that have been pilot tested in previous research and are designed to minimize sampling bias. Interested families will be screened for project eligibility and, if eligible, consented/assented into the study and scheduled to complete the baseline assessment.

## Assignment of interventions: allocation

### Sequence generation {16a}

A computer program will be used to generate a randomly permutated scheme to allocate subject identification (ID) numbers to the experimental and passive control conditions in a 1:1 ratio. The principal investigator (PI) for the study will generate the allocation sequence prior to study enrollment.

### Concealment mechanism {16b}

The a priori-generated allocations will be placed in sealed envelopes, which will be labeled with a unique ID number corresponding to the IDs to be assigned to participating dyads at enrollment.

### Implementation {16c}

After completion of the baseline survey, data collectors will open the envelope associated with the dyad’s participant ID number to reveal the study condition assigned to the dyad. After the baseline, CHWs will reach out to families enrolled in the experimental arm to schedule their first FTT+ intervention session via Zoom.

## Assignment of interventions: blinding

### Who will be blinded {17a}

All data collection staff (assessors) will be blinded.

### Procedure for unblinding if needed {17b}

A procedure for unblinding will not be needed, as participants will be aware of which condition they are assigned to immediately after they complete the baseline survey.

## Data collection and management

### Plans for assessment and collection of outcomes {18a}

Data will be collected at three time-points: baseline, 3 months post-baseline, and 9 months post-baseline. Data will be collected using self-report, pen and paper surveys in the participants’ homes or an agreed upon private location in the community. There will be no usage of a proxy respondent in data collection activities. The surveys will be written at a 4^th^ grade reading level and will be available in either English or Spanish, based on participant preference. The investigative team will have experience in the development and implementation of bilingual (English/Spanish) and psychometrically sound measurement protocols. Surveys will be translated using a forward–backward translation method. Two translators will independently translate the surveys, then a third translator will resolve any differences between the two. The measures utilized in the survey will have good internal consistency (α > .7) and have been previously used, to good effect, in the South Bronx with the target populations. During the first year of the project, we will finalize the quantitative measurement protocol. To ensure linguistic and cultural appropriateness, we will also pilot test the measures with the target population.

Prior to the completion of each survey, data collection staff will reiterate the importance of truthful responses and will reinforce that all data will be kept confidential. Data collection staff will be available to answer any participant questions during survey completion.

At baseline, we will collect demographic data (race, ethnicity, age, educational attainment of parents, etc.) in order to establish baseline equivalence of the analytic sample. We will also assess outcomes at baseline. At the immediate (3-month) and delayed (9-month) follow-ups, we will assess outcomes, mediator variables, and measures of intervention dosage/contamination.

### Plans to promote participant retention and complete follow-up {18b}

For all data collection activities, we will provide flexible scheduling based around the participants’ availability, including during evening hours and on weekends. Study staff will call prior to each appointment to remind participants about their scheduled sessions. Data collectors will update contact information at each visit in order to maintain accurate tracking information, thus increasing the likelihood that the family will be reachable and continue participating. Further, participants will be asked at enrollment to provide contact information for three people who know how to reach them in most circumstances. These methods were used in previous work in the South Bronx with an attrition rate of 8.6% over 12 months [23]. Prior experience indicates that virtually all respondents will have a cell phone or access to a phone. However, as part of the consent process, we will obtain permission to revisit the home to make follow-up appointments if phone contact is not possible.

All participants will be invited to continue data collection activities regardless of whether they deviate from or discontinue intervention protocols. All outcome data will be collected from participants who discontinue or deviate from the intervention protocols. To minimize missing data, participants will be reassured of the confidential status of their responses. Additionally, staff will review each question in the survey after completion to offer an additional opportunity for participants to respond should they leave a question unanswered.

### Data management {19}

Pen and paper self-report surveys will be administered by trained data collection staff. Completed survey packets will be stored in locked file cabinets in the CLAFH Manhattan study office. Any documents containing participants’ names will be kept in a separate location. Data from the paper surveys will be entered into an electronic, password-protected database by trained data entry staff. The evaluation coordinator will review >10% of all entered survey data to ensure accuracy.

### Confidentiality {27}

To protect the integrity of the participants’ data, the following procedures will be followed. First, all participants enrolled in the study will be assigned an ID number. This code number will be used on all information collected from participants, including consent forms and surveys. Participants will never be identified by name on consent forms or surveys. No other information that would reveal the participants’ identity will be found on the materials collected. We will maintain a list of participants with links between identifying information and IDs to which only the Local Evaluator and the Project Director will have access. The lists will be kept on a password-protected computer system, separate from all other data files. All data will be stored in locked file cabinets or on password-protected computers and databases. The study office is in a building monitored by security staff as well.

### Plans for collection, laboratory evaluation, and storage of biological specimens for genetic or molecular analysis in this trial/future use {33}

There are no plans for collection, laboratory evaluation, or storage of biological specimens for genetic or molecular analysis in this trial.

## Statistical methods

### Statistical methods for primary and secondary outcomes {20a}

SAS, M Plus, and/or SPSS statistical software will be used for data analysis. Statistical significance will be assessed at *p*<0.05. In addition to traditional hypothesis testing, we will also utilize the Bayesian Interpretation of Estimates Framework to evaluate intervention effects [32].

We will use an intent-to-treat (ITT) analysis of the 9-month outcomes and single degree of freedom contrasts comparing the intervention to the control group in primary outcomes (i.e., sexual debut/ever sex) and secondary outcomes (i.e., frequency of sex, lifetime number of sexual partners, number of unprotected sex acts, and adolescent linkage to health and educational/vocational services in the community). For dichotomous outcomes (e.g., ever sex), a Bayes-adjusted confidence interval approach or bootstrapped method will be used. Alternatively, logistic regression with the treatment condition represented by two dummy variables will be pursued, allowing us to introduce covariates into the system. For count variables (e.g., frequency of sex), contrasts will use either Poisson regression or negative-binomial regression (or zero-inflated variants of them) whereby the treatment condition is represented in the equation by two dummy variables. If the count outcomes do not conform to the presumed distributional properties of these methods, then linear regression with some form of bootstrapping will be pursued as an alternative. For continuous variables (e.g., number of unprotected sex acts) linear regression with the treatment as dummy variables will be pursued.

To address the possibility of multiplicity error, we will evaluate the coefficient alphas and factor structures of all our multi-item measures to ensure that they are behaving in a way that one would expect based on their psychometric histories. Some of the constructs in our conceptual model reflect variable categories with multiple variables or dimensions. We will routinely examine the intercorrelations of variables and, also applying substantive criteria and the results of confirmatory factor analyses, make decisions about combining indices or introducing latent constructs into the analysis. At times, we will conduct multiple significance tests, and there will be concern for inflated experiment-wise error rates based on how the family of contrasts is defined. We will compare the robustness of our conclusions both with and without statistical corrections for multiple tests. In general, we will use a Holm-adjusted modified Bonferroni method or the false discovery rate method [33] to control experiment-wise error rates, both of which are more powerful than the Bonferroni methods.

### Interim analyses {21b}

There will not be any interim analysis on this project.

### Methods for additional analyses (e.g., subgroup analyses) {20b}

#### Subgroup analyses

We will conduct subgroup analyses to evaluate whether intervention effect differs across three factors: adolescent ethnicity/race, gender, and age. Specifically, subgroup analyses will be used to evaluate if intervention effects differ between Latino and Black adolescents, male and female adolescents, and younger (12–14-years-old) and older (15–17-years-old) adolescents. We hypothesize that no statistical difference will be found between these subgroups and that the intervention will be equally efficacious for each. When performing subgroup analyses, we will use a regression analysis and inclusion of an interaction term between intervention assignment and potential moderating factors in the model.

### Methods in analysis to handle protocol non-adherence and any statistical methods to handle missing data {20c}

In some of the analyses, there is a chance of missing data due to participants not answering every question in the surveys. Data may be missing at random (MAR), missing completely at random (MCAR), or missing in a systematic way (systematically). First, we prepare for this by using a dummy variable to evaluate whether data are missing or not. Then, we look to see if the missing data are predicted by other variables of interest. We will apply Little’s multivariate test for MCAR [34]. Data can also be missing due to loss of follow-up. We will evaluate missing data, those questions that are not answered, and those lost at follow-up independently of each other. Depending on what data are missing, we will approach such data using full information maximum likelihood methods, multiple imputation methods, or systematic model missing data bias [35].

### Plans to give access to the full protocol, participant-level data, and statistical codes {31c}

We plan to publish the full trial protocol to make it widely available to other researchers. However, participant-level data and statistical codes will not be made available to protect participant confidentiality. Due to the sensitive nature of the data and the facts that participants are recruited from a small geographical area and we are collecting demographic data (at baseline) that may threaten the confidentiality of participants, access to participant-level data will not be released publicly. However, we will make the data available for a 5-year period starting 3 months after article publication for investigators whose proposed use of the data has been approved by an independent review committee to conduct analyses to achieve the aims in their proposal. To gain access, data requesters will need to sign a data access agreement.

## Oversight and monitoring

### Composition of the coordinating center and trial steering committee {5d}

There will be no formal coordinating center or trial steering committee for the current study. The study will be coordinated by the project manager and lead evaluator, who will each report to the PI, Dr. Vincent Guilamo-Ramos. All activities will be implemented using two teams: the data collection and intervention teams. The lead evaluator (MTK) will coordinate day-to-day data collection, entry, and analysis activities. Meanwhile, the project manager (AB) will oversee day-to-day intervention activities and quality improvement methods for intervention delivery, namely through independent observations of recorded sessions and biweekly training of intervention staff. Dividing the two teams will allow for blinding of data collectors, decreased likelihood of social desirability bias, and reduced risk of contamination.

### Composition of the data monitoring committee, its role and reporting structure {21a}

No data and safety monitoring board (DSMB) will be convened for this study. A DSMB will not be necessary as there are no anticipated risks to participant safety or to the integrity of data.

### Adverse event reporting and harms {22}

All participants will be provided with a copy of the consent form that includes contact information for the PI and the Duke Health IRB. Participants can use this contact information to report adverse events or unanticipated problems. Possible adverse events that occur will be brought to the attention of the PI, who will notify the Duke Health IRB immediately. The Duke Health IRB will determine whether it is appropriate to stop research activities temporarily or provide suggestions/make modifications to the protocol. Possible modifications include adding possible adverse events to the consent forms and re-consenting all data collection participants. We do not anticipate any negative effects of participating in our survey.

### Frequency and plans for auditing trial conduct {23}

An annual audit will be conducted to protect the integrity of all collected data as part of this trial. In addition, participant data will be kept beyond the term of the study, for up to 6 years, and will need to be monitored.

The PI will ensure that data collection is conducted and reported according to the protocol. There will be annual auditing trials to ensure the protocol is followed, there are no issues with the informed consent procedure, and record keeping is accurate. Specifically, the lead evaluator and PI will conduct semi-annual field visits to assess that procedures are being implemented as planned at all sites.

### Plans for communicating important protocol amendments to relevant parties (e.g. trial participants, ethical committees) {25}

Protocol amendments will need to be reviewed and approved by the IRB. Any major changes to the trial will have to be approved by the sponsor, namely ACF. Furthermore, all changes to the protocols will be reflected and updated on clinicaltrials.gov.

### Dissemination plans {31a}

The study’s results will be disseminated via peer-reviewed journals, presentations at national conferences, and media/press releases.

## Discussion

Adolescent sexual health remains a national public health priority, and adolescents represent a priority population for sexual health promotion, as outlined in the National HIV Strategic Plan, the National STI Strategic Plan, the U.S. Department of Health and Human Services Teen Pregnancy Prevention Program, and the recent NASEM report [7, 28, 36, 37]. Research suggests that prevention programming is not only a successful method of reducing sexual risk behavior, but also a way to mitigate billions spent annually on teen pregnancies and STIs. Each year in the U.S., the annual federal and state cost of teen pregnancies is estimated at $9.4 billion [38]. The lifetime medical costs of incident STIs among youth total $4.2 billion [39]. Furthermore, each new HIV infection, alone, has been estimated to result in the accumulation of more than $400,000 in lifetime medical costs [6, 40]. Efficacious and scalable programs to promote adolescent sexual health, particularly of those at the greatest risk of negative sexual health outcomes, namely Latino, Black, and older adolescents, are urgently needed.

We propose to implement, evaluate, and scale up the online parent-based intervention, FTT+, to reduce adolescent sexual risk behavior. FTT+ will address specific gaps in the cadre of available parent-based interventions, including (1) in its adaptation to address the role of the parent in shaping both younger and older adolescent sexual health; and (2) its utilization of a teleconference application (e.g., Zoom) as a delivery mechanism to facilitate broad reach and scale-up.

Certain limitations will warrant consideration when data collection is complete and the study findings are interpreted. First, each assessment will rely on self-report data, which may be prone to recall and social desirability bias. However, we will include a social desirability index, as well as psychometrically sound measurement and data collection procedures, that we have successfully used in prior studies with the sample population. Second, we will be recruiting participants from a single geographic setting, namely the South Bronx. Therefore, caution is warranted when making inferences to populations and contexts that differ markedly from the proposed sample.

Taken together, the remote parent-based intervention delivery and use of a teleconferencing application (i.e., Zoom) to deliver it constitute an innovative and comparatively low-cost option for broad scale-up and dissemination of sexual health programming to families. If efficacious, the adaptation and scale-up of the FTT intervention will represent an opportunity to enhance the reach and adoption of parent-based approaches in addressing adolescent sexual health in the U.S.

### Trial status

More than half of the sample have consented and enrolled on our study (>375 dyads), and we are actively conducting 3- and 9-month data collection activities with enrolled families. Recruitment began in March 2021 and is ongoing. The protocol was submitted to and accepted by ClinicalTrials.gov prior to the start of recruitment in February 2021. No significant changes were made to the protocol after the initial submission. The anticipated end date of recruitment for this study is May 2023.

## Data Availability

The project team will share all individual participant data that underlie the results reported in any future published manuscript after deidentification (text, tables, figures, and appendices). The project team will make the data available for a 5-year period beginning 3 months after article publication for investigators whose proposed use of the data has been approved by an independent review committee to conduct analyses to achieve the aims in their proposal. To gain access, data requesters will need to sign a data access agreement.

## Abbreviations

ACF: Administration for Children and Families
CHW: Community health worker
CLAFH: Center for Latino Adolescent and Family Health
COVID-19: Coronavirus disease 2019
DSMB: Data and safety monitoring board
DUSON: Duke University School of Nursing
FTT+: Families Talking Together Plus
HHS: U.S. Department of Health and Human Services
HIV: Human immunodeficiency virus
HRSA: Health Resources and Services Administration
ID: Identification
IRB: Institutional Review Board
ITT: Intent-to-treat
MAR: Missing at random
MCAR: Missing completely at random
NASEM: National Academies of Sciences, Engineering, and Medicine
NYC: New York City
PI: Principal Investigator
RCT: Randomized controlled trial
SRH: Sexual and reproductive health
STI: Sexually transmitted infection
U.S.: United States
